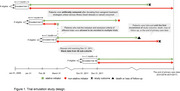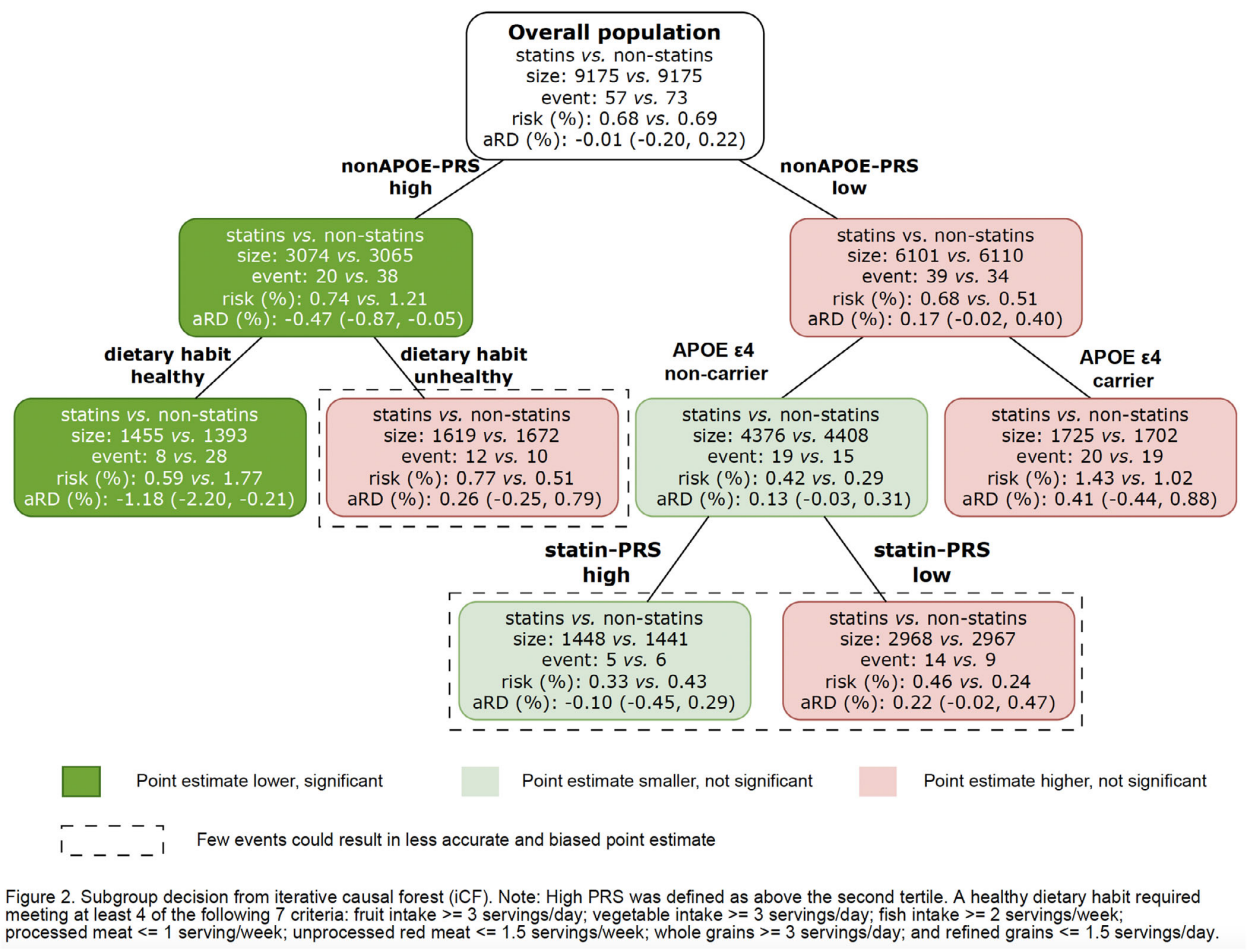# Integrate Real‐World Data and Genetics: a causal machine learning approach to assess Statin’s Effect on Incident Dementia

**DOI:** 10.1002/alz70859_098710

**Published:** 2025-12-25

**Authors:** Tiansheng Wang, Yongjie Lai, Elizabeth C. Mormino, Guorong Wu, Gwenn A Garden, Til Stürmer, Yang Xu

**Affiliations:** ^1^ Universtiy of North Carolina at Chapel Hill, Chapel Hill, NC USA; ^2^ Peking University School of Pharmaceutical Sciences, Beijing, Beijing China; ^3^ Stanford University School of Medicine, Stanford, CA USA; ^4^ University of North Carolina at Chapel Hill, Chapel Hill, NC USA

## Abstract

**Background:**

The impact of statins on dementia has been debated for over two decades. Population differences may modify statins effects, contributing to inconsistent findings. Approaches to identify patients most likely to benefit are needed. We aim to apply iterative causal forest (iCF) causal machine learning algorithm in emulated trials in UK Biobank (UKB) to assess heterogeneous treatment effects (HTEs) of statins on incident dementia.

**Method:**

We conducted an emulated target trial using 36 monthly sequential trials (2009–2011) with linked UKB primary care data comparing statins initiators and non‐initiators aged 55+. Incident dementia was identified using UKB’s algorithmically‐defined outcomes and Read Version 2 codes. Patients were followed from baseline until dementia, death, loss to follow‐up, or end of primary care (5/31/2016–8/31/2017). Confounders and potential effect modifiers include genetics, socioeconomic status, lifestyle, comorbidities, and medications. Genetic modifiers included Apolipoprotein E (APOE) genotype, the polygenic risk score (PRS) for Alzheimer’s disease excluding the APOE region (nonAPOE‐PRS), and the PRS for statins response predicting low‐density lipoprotein cholesterol reduction after statins use (statin‐PRS). We performed propensity score (PS)‐matching on the 36 (stacked) sequential trials, censored upon treatment deviation in per‐protocol analysis, accounting for selection using inverse probability of censoring weights (IPCW). Next, we applied iCF to PS‐matched trials to identify subgroups with HTEs, and estimated 5‐year adjusted risk difference (aRD) by inverse probability treatment and censoring weights in each identified subgroup.

**Result:**

The overall aRD was ‐0.01% (‐0.20% to 0.22%), risks of 0.68% vs. 0.69%, in PS matched cohort (9175 statins initiators vs 9175 non‐statins initiators). iCF revealed HTEs (*P*‐value = 0.001) and identified patients with a high [> second tertile] nonAPOE‐PRS and healthy dietary habits (³ 4 of 7 criteria, see Figure 2. legend) benefit most from statins (aRD ‐1.18% [‐2.20% to ‐0.21%], risk 0.59% vs 1.77%), while those with high nonAPOE‐PRS alone exhibited a smaller benefit (aRD ‐0.47% [‐0.87% to ‐0.05%], risk 0.74% vs 1.21%).

**Conclusion:**

Our emulated trial indicated that statins did not demonstrate a protective effect against incident dementia in overall population. However, a subpopulation with high nonAPOE‐PRS (which predicts AD dementia) and healthy dietary habits may benefit from statins use.